# Digital Interventions for Treating Post-COVID or Long-COVID Symptoms: Scoping Review

**DOI:** 10.2196/45711

**Published:** 2023-04-17

**Authors:** Robin Rinn, Lingling Gao, Sarah Schoeneich, Alina Dahmen, Vinayak Anand Kumar, Petra Becker, Sonia Lippke

**Affiliations:** 1 Constructor University Bremen Germany; 2 Julius-Maximilians-Universität Würzburg Germany; 3 Klinikum Wolfsburg Wolfsburg Germany; 4 Dr Becker Klinikgruppe Cologne Germany

**Keywords:** post-COVID/long-COVID symptom recovery, postacute COVID-19 symptoms, treatment, therapy, mHealth, mobile health, rehabilitation, COVID-19

## Abstract

**Background:**

Patients with post-COVID/long-COVID symptoms need support, and health care professionals need to be able to provide evidence-based patient care. Digital interventions can meet these requirements, especially if personal contact is limited.

**Objective:**

We reviewed evidence-based digital interventions that are currently available to help manage physical and mental health in patients with post-COVID/long-COVID symptoms.

**Methods:**

A scoping review was carried out summarizing novel digital health interventions for treating post-COVID/long-COVID patients. Using the PICO (population, intervention, comparison, outcome) scheme, original studies were summarized, in which patients with post-COVID/long-COVID symptoms used digital interventions to help aid recovery.

**Results:**

From all scanned articles, 8 original studies matched the inclusion criteria. Of the 8 studies, 3 were “pretest” studies, 3 described the implementation of a telerehabilitation program, 1 was a post-COVID/long-COVID program, and 1 described the results of qualitative interviews with patients who used an online peer-support group. Following the PICO scheme, we summarized previous studies. Studies varied in terms of participants (P), ranging from adults in different countries, such as former hospitalized patients with COVID-19, to individuals in disadvantaged communities in the United Kingdom, as well as health care workers. In addition, the studies included patients who had previously been infected with COVID-19 and who had ongoing symptoms. Some studies focused on individuals with specific symptoms, including those with either post–COVID-19 or long-term symptoms, while other studies included patients based on participation in online peer-support groups. The interventions (I) also varied. Most interventions used a combination of psychological and physical exercises, but they varied in duration, frequency, and social dimensions. The reviewed studies investigated the physical and mental health conditions of patients with post-COVID/long-COVID symptoms. Most studies had no control (C) group, and most studies reported outcomes (O) or improvements in physiological health perception, some physical conditions, fatigue, and some psychological aspects such as depression. However, some studies found no improvements in bowel or bladder problems, concentration, short-term memory, unpleasant dreams, physical ailments, perceived bodily pain, emotional ailments, and perceived mental health.

**Conclusions:**

More systematic research with larger sample sizes is required to overcome sampling bias and include health care professionals’ perspectives, as well as help patients mobilize support from health care professionals and social network partners. The evidence so far suggests that patients should be provided with digital interventions to manage symptoms and reintegrate into everyday life, including work.

## Introduction

### Background

Doctor O is a medical doctor who sees many patients having post-COVID/long-COVID symptoms and experiencing recovery difficulties. She has no knowledge of sustainable treatments and therapies to help patients recover from post-COVID/long-COVID symptoms. Her patients, who were previously infected with COVID, need efficacious symptom management. Health care workers like Doctor O, in turn, require access to evidence-based treatment options. To address both objectives, digital interventions are expected to support face-to-face treatment, especially if personal contact is limited due to time constraints or because patients fear infection or reinfection, live far away, or cannot commute due to their health limitations. Furthermore, digital interventions seem to be helpful because they can bridge the undersupply of health care and are continuously available. Thus, evidence about the extent to which digital interventions are feasible and effective for patients with post-COVID/long-COVID symptoms is crucial.

Digital interventions are promising in this respect [1], as they have been found to be effective in other areas, such as psychosomatic rehabilitation [2], asthma self-care [3], coping, and fatigue due to the effects of cancer [4]. Furthermore, digital interventions can be used to improve health in people living with frailty [5]. However, there are limited interventions that have been scientifically evaluated in patients with post-COVID/long-COVID symptoms. The goal of this review is to summarize the current evidence on digital interventions that aim to help patients with post-COVID/long-COVID symptoms in terms of improvements in their physical and mental health or well-being. Thus, we aim to provide recommendations based on the current literature to inform further research concerning post-COVID/long-COVID–specific digital interventions in the future and to inform health care professionals for developing future post-COVID/long-COVID–specific digital interventions.

### Post-COVID/Long-COVID Symptoms and Current Issues

Since the exact mechanisms that cause post-COVID/long-COVID symptoms are still unknown, there are only a few evidence-based interventions. Patients are treated symptomatically rather than causally [6,7], and symptoms are usually treated by a range of specialists [8]. A negative side effect of this approach is that such treatments make it difficult for general practitioners and health care workers to treat patients as they routinely do. Patients might experience unmet expectations, which can cause a feeling of not being well understood [9,10]. This has detrimental consequences because in some cases, desperate patients are more likely to listen to the advice of uneducated people on social media, use nonvalidated information from the internet, use the wrong medications, or overdose on medications in the hope of getting better quickly [9-12].

Evidence-based digital health interventions can therefore be helpful for post-COVID/long-COVID patients because they have the potential to increase access to care, are cost-effective, have a low threshold, and are scalable for large patient groups [2,6]. Digital interventions therefore not only address time and resource constraints, but can also treat various manifestations of post-COVID/long-COVID symptoms. Furthermore, such interventions can be synchronous or asynchronous and therefore offer a variety of different approaches [13,14]; the former includes real-time and usually face-to-face contact, for example, via videoconferencing software or real-time chat [15,16].

A previous literature review focused on telerehabilitation in people with post-COVID/long-COVID symptoms with a focus on dyspnea symptoms and found that digital interventions may alleviate symptoms compared with no intervention [17]. The aforementioned study contributes partly to the research question of this paper. However, besides telerehabilitation and specific symptoms, it is still unknown which digital interventions can help patients to recover from other post-COVID/long-COVID symptoms. This paper therefore addresses this gap and includes a broader spectrum of symptoms beyond dyspnea and the unrefined characteristics of functional performance [17-20]. Accordingly, the research question is as follows: What evidence-based digital interventions exist to help manage physical and mental health in patients with post-COVID/long-COVID symptoms?

## Methods

### Research Question

Our detailed research question is as follows: Which digital interventions help post-COVID/long-COVID patients to recover in terms of their physical and mental health or mental well-being? According to the PICO (population, intervention, comparison, outcome) scheme [21], we will address the current state of the literature. We will then develop options to improve future (research on) digital interventions aiming to promote health and well-being in post-COVID/long-COVID patients while discussing the current limitations.

### Protocol and Preregistration

This research was not explicitly preregistered [22]. However, the review is part of an ongoing research project on the treatment and diagnosis of post-COVID/long-COVID symptoms that has been preregistered [8].

### Definitions of Central Concepts

For this research, post-COVID/long-COVID symptoms were defined as persistent, new-onset, or worsening symptoms after an acute COVID-19 infection. There were no restrictions on how COVID-19 infections or the persistence/worsening of symptoms were diagnosed. Furthermore, a digital intervention was defined as a treatment accessible by individuals via a mobile phone, a computer, or other devices with internet access. The treatment could be delivered either synchronously or asynchronously [15] or via mixed methods by health care professionals, scientists, or nonprofessionals (eg, in the form of self-help groups on social media) [23]. The most important aspect was that patients received a digital treatment that was sufficiently described and scientifically evaluated. Only digital interventions playing a major role in the treatment of post-COVID/long-COVID symptoms were considered, that is, we did not include interventions where digital techniques were used (optionally) to inform participants. We also took research without control groups into account. Compared to other authors, we did not limit our search to telemedicine or telerehabilitation [17].

### Systematic Approach and Search Terms

The review was carried out systematically and with an *a priori* design. Five reviewers screened the titles and abstracts. Papers that appeared relevant from their titles and abstracts were reviewed as full-text papers to check whether they met the inclusion criteria. Because we wanted to find a wide range of literature from different scientific fields, we decided to use a search strategy that covered as many different terms as possible, rather than a search strategy in which one repeatedly uses one term with different punctuations (eg, parentheses, slashes, etc) or words, such as “and” and “or.” Specifically, the following method was used. We used each of the following 5 terms for postacute COVID-19 syndrome: (1) long-COVID, (2) post-COVID, (3) long haulers, (4) PACS, and (5) postacute COVID-19 syndrome, and combined them with each of the following 11 search terms: (1) intervention, (2) training, (3) therapy, (4) rehabilitation, (5) mHealth, (6) eHealth, (7) digital intervention, (8) app, (9) web app, (10) digital, and (11) recovery (eg, one search term was long COVID + digital intervention). Thus, in each database that we used (Proquest, Ebsco, PubMed, PubPsych, and Scopus), we searched for 55 different terms. The search took place between May and July 2022. Literature was reviewed with consistent search terms and screening rules, in parallel, across different databases. Reviewers responsible for each database removed duplicates found within each database and screened each publication for inclusion.

Although duplicates between databases were not removed, prior to title and abstract screening, the final list of papers included was reviewed and duplicates were removed at this later stage. The approach adopted does overestimate the number of publications pertaining to post-COVID/long-COVID symptoms. However, we validated that the search strategy achieved a high coverage rate of publications in the target topic. Further efforts were made to ensure coverage of the literature; in August 2022, the literature section from each paper was reviewed to search for further studies that fit the inclusion criteria. Moreover, expert interviews were carried out and relevant social media accounts were screened, leading to 5 further studies being screened.

### Inclusion Criteria

To be included in the review, scientific work had to be original studies and published in 2021 and 2022. Moreover, only English articles in academic journals were included. No restrictions were placed on the methods used. Both qualitative and quantitative analyses were accepted, and observational studies were included if the target group was provided a digital health intervention. Finally, studies had to evaluate the outcome of digital health interventions. Study protocols, reviews, periodicals, etc, as well as simple monitoring studies were therefore not included.

## Results

**Figure 1:**
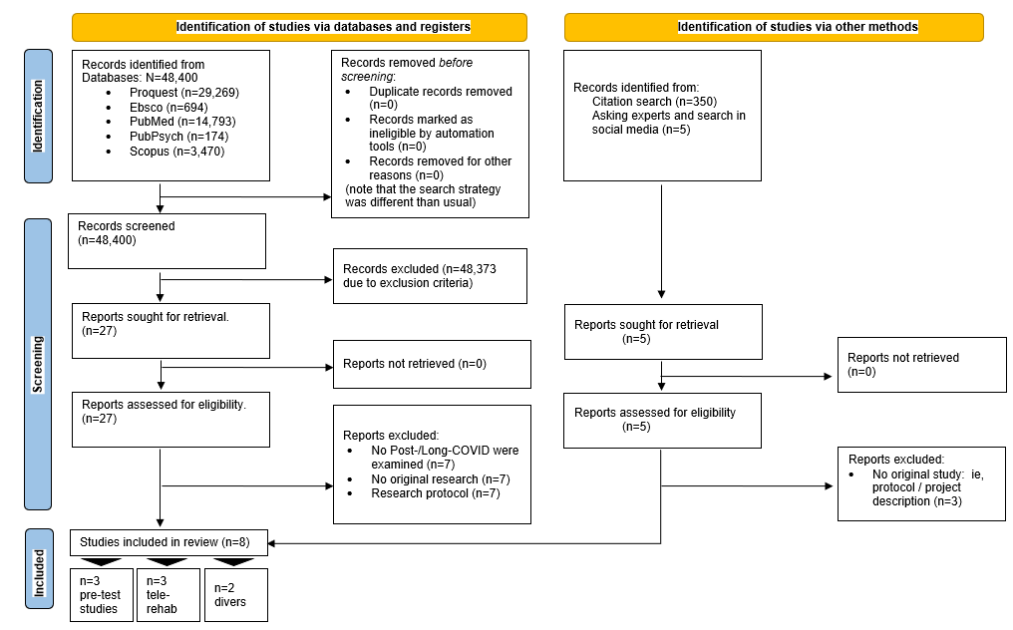
PRISMA (Preferred Reporting Items for Systematic Reviews and Meta-Analyses) flow chart.

An overview of the screening procedure can be found in the PRISMA (Preferred Reporting Items for Systematic Reviews and Meta-Analyses) flow chart (Figure 1), and a summary can be found in Table 1. In total, 8 original studies matching the inclusion criteria were found. Three of those can be considered as “pretest” studies, since they examined the feasibility and applicability of the interventions with few participants (N<27) [4,20,24]. Moreover, 3 articles described the implementation of a telerehabilitation program [25-27], 1 involved a post-COVID/long-COVID program [28] with a seminar (1-hour session per week for 7 weeks) where participants learned about coping with post-COVID/long-COVID symptoms, and 1 described the results of qualitative interviews with patients who used post-COVID/long-COVID online peer-support groups [29]. Because of the inconsistent methods and data collected, a scoping review format was chosen.

**Table 1:** Summary of the literature review.

Source	Participants and country	Setting and study type	Intervention	Outcome variables	Outcomes
Pretest study [20]	27 (21 for analyses) individuals with persisting respiratory symptoms after a COVID-19 infection (>28 days).	Study was conducted in Ireland. The intervention took place online via Zoom. Single arm intervention study with pre-post comparison.	10 weeks, bi-weekly vocal and breathing sessions for 45 min. Live online training program. The program consisted of mindfulness, body scan, and relaxation trainings, as well as breathing and singing.	Pre-COVID health conditions Information about the COVID infection C19-YRS^a^ PTSD^b^ DSQ-SF^c^ Semistructured interviews to assess the impact and experience of the intervention.	Participants showed significant pre- to postintervention improvements in breathlessness symptoms. There was a decrease in the number of patients who fulfilled the criteria for myalgic encephalomyelitis and chronic fatigue syndrome. Participants reported positive experiences with the intervention.
Telerehabilitation [25]	150 (115 participants who completed the intervention) individuals with persistent dyspnea symptoms after a COVID-19 infection.	Online in cooperation with 7 health care centers in San Bernardo, Santiago, Chile. Quasiexperimental pre-post intervention study.	Telerehabilitation program. 24 sessions over 9 weeks (2-3 per week, approximately 45 min each). Program includes warm-up, breathing exercises, stretching, aerobic exercises, and strength exercises. Program was set to moderate intensity and was tweaked a little according to each patient’s condition. Supervision was done by a physiotherapist via telephone.	Physical capacity (measured by participant ability to perform in the 1-min sit-to-stand test). Modified Borg Scale to measure dyspnea and fatigue. Pulse, oxygen saturation, and heart rate. SF-36^d^ HRQoL^e^	Physical capacity, HRQoL, dyspnea, and fatigue improved after the intervention. Analysis of patients who were nonhospitalized versus hospitalized showed improvements in all outcomes, except fatigue. In the analysis of patients who were admitted to the intensive care unit versus not, the results showed that all outcomes improved, except for 4 dimensions of quality of life (body pain, general health perceptions, mental health, and emotional role limitations).
Interview [29]	11 individuals who suspected that they had long COVID.	United Kingdom–based and part of online social media peer-support groups. Observational study without pre-post comparison.	Retrospective cross-sectional observational study. Participants who took part in existing long-COVID (self-help) peer-support groups on social media were interviewed.	Qualitative method using semistructured interviews. Interviewer asked why participants joined the group, their experiences, and how they feel about their role in the group. Impact the groups have on patients and their experiences within these spaces. Identify ways online peer-support groups can be situated within broader long-COVID recovery planning.	Among other things, the peer-support groups helped participants: To get recognition and to be taken seriously. To get new information for treatment options although they were only partly evidence based. To find social connections, to feel less lonely, and to improve their mental well-being.
Pretest study [24]	10 (8 took part in all measurement time points) participants with persisting long-COVID symptoms. Patients resided in deprived communities and received a GBP 30 (approximately US $40.50) voucher for participation.	Online in the United Kingdom. Single-arm intervention study with pre-post comparison.	Intervention based on sport and exercise medicine, and functional rehabilitation to assist people with long COVID. Comprised 3 online meetings with a multidisciplinary team in a virtual clinic via Zoom (approximately 1 hour each). Advice to gradually increase physical activity. Pacing advice. Monitoring of symptoms.	QOL-5D^f^ FSS^g^ PCFSS^h^ CARE Measure GAD^i^ PHQ9^j^ PREM^k^	Among all participants, there were improvements in pre- and postmeasurements, especially in participants’ self-reported fatigue, pain, and anxiety.
Long-COVID care [28]	149 individuals (76 took part in pre-post intervention measurements). Self-diagnosed long-COVID patients in line with the National Institute for Health and Care Excellence guidelines. Patients had to experience long-term symptoms of COVID-19. Patients were social, health, and care staff.	Online in the United Kingdom via Microsoft Teams. Single arm intervention study with pre-post comparison.	7 weeks online, 1 session per week for 1 hour. Rehabilitation course. Intervention included a workbook to develop new skills such as self-monitoring, action planning, and problem solving. Exercises included relaxation, breathing, information about sleep, mindfulness, nutrition, stress management, energy conservation, and activity management.	EQ-5D-5L HRQoL Overall health self-report via a visual analog scale (VAS).	Overall improvement in health over time from before to after the course, but only 3% of the participants reported being back to their pre-COVID health state. Another pre-post comparison revealed that 30%-50% of the participants reported that their mobility, self-care, usual activities, pain/discomfort, and anxiety/depression improved over time (between before and after the course).
Telerehabilitation [27]	35 individuals took part in the main intervention (22 fully completed the exercise program). Formerly hospitalized for longer than 7 days. 4-6 weeks after hospitalization. Moderate COVID-19 infection during hospitalization. Fatigue is the main symptom at discharge.	Greek patients who were hospitalized and who were given the advice to self-isolate at home. Single-arm intervention study with pre-post comparison.	2 stages Stage 1: four 1-hour teleconference sessions with a health care professional (learning how to use a specifically designed eBook and talking about physical and emotional status, diet, and quality of life). Stage 2: 2-month exercise program with daily self-practice. 5-10 min warm up, 15-20 min main workout, and 5-10 min cool down. Breathing, healthy eating, physical exercise, and relaxation. Telerehabilitation session every 10 days.	Physical activity (IPAQ-Gr^l^) SF-36 Anxiety and depression (HADS^m^) Short Physical Performance Battery (SPPB) 60-s sit-to-stand test (60secSTS) 3-min step test (3MST) SpO_2_ (oxygen saturation)	People managed to perform more repetitions in the 60-s sit-to-stand test, which indicates an improvement in the physical fitness of participants. There were several improvements in the SF-36 questionnaire, especially in the subtests that examine the physical and mental health component, as well as the subtests for physical role. For social functioning as well as for general health vitality, the researchers observed improvements. There was an improvement in the HADS test, indicating less anxiety and depression after the intervention compared to before the intervention.
Telerehabilitation [26]	120 (105) formerly hospitalized individuals who still experience dyspnea complaints.	Three hospitals from 2 provinces in China. Randomized controlled trial.	Unsupervised home-based exercises. 6-week exercise. Program delivered via a smartphone and remotely monitored with heart rate telemetry. Teleconsultation with therapists once per week. 3-4 sessions per week: breathing control, aerobic exercise, and lower limb muscle strength (LMS) training.	Functional exercise capacity at posttreatment measured with the 6-min walking test (6MWT) in meters. Lung function: LMS, FEV1^n^, FVC^o^, PEF^p^, and MVV^q^ HRQoL SF-12^r^ (including PCS^s^ and MCS^t^) Perceived dyspnea via the mMRC^u^ scale.	For the 6-min walking distance measurement, the mean of the control group increased by 17.1 m from baseline to posttreatment, whereas the mean of the intervention group improved by 80.2 m. HRQoL and LMS improved in the intervention group. Pulmonary function improved in both groups. 90.4% were dyspnea free in the intervention group compared to 61.7% in the control group, but there were no long-term effects.
Pretest study [4]	7 women who still experience fatigue due to a COVID-19 infection.	An app was used, and interviews took part online in Austria. Single-arm intervention study with pre-post comparison.	The used app was originally built to help fight fatigue in cancer patients, but it has been tested to see whether it would apply to long-COVID patients. The program consists of education, physical activities, and support (videos, tutorials, etc) to help users identify thoughts, behaviors, and symptoms affecting energy levels. Participants used the app for more than 2 weeks (at least 3 times a week for more than 20 min).	Qualitative study with semistructured interviews. User experience (functions of the app). Helpful self-management strategies. Improvement suggestions.	Overall user experience was good, with users claiming it was easy to use. Participants found the intervention useful in terms of relaxation exercises and energy measurement. Many reported learning new techniques.

^a^C19-YRS: COVID-19 Yorkshire Rehab Screen.

^b^PTSD: questions about posttraumatic stress disorder.

^c^DSQ-SF: DePaul Symptom Questionnaire, Short Form.

^d^SF-36: 36-Item Short-Form Health Survey.

^e^HRQoL: health-related quality of life.

^f^QOL-5D: European Quality of Life 5 Dimensions Version.

^g^FSS: Fatigue Severity Scale.

^h^PCFSS: Post-COVID-19 Functional Status Scale.

^i^GAD: Generalized Anxiety Disorder Assessment.

^j^PHQ9: Patient Health Questionnaire-9.

^k^PREM: Patient-Reported Experience Measure.

^l^IPAQ-Gr: International Physical Activity Questionnaire: Greek version.

^m^HADS: Hospital Anxiety and Depression Scale.

^n^FEV1: one-second capacity.

^o^FVC: forced vital capacity.

^p^PEF: peak expiratory flow.

^q^MVV: maximum voluntary ventilation.

^r^SF-12: 12-Item Short-Form Health Survey.

^s^PCS: Physical Component Summary.

^t^MCS: Mental Component Summary.

^u^mMRC: modified British Medical Research Council.

### Participants

The included studies dealt exclusively with adults from different countries. Specific subgroups comprised former COVID-19 hospitalized patients; individuals in medically underserved areas; and social, health, and care staff in the United Kingdom.

Specifically, in the 3 pretest studies, 27 (adults from Ireland) [20], 10 (adults living in underserved communities in the United Kingdom) [24], and 7 (adults in Austria) [4] participants took part, and from pre- to postintervention, 5 [20], 2 [24], and 0 [4] participants dropped out. Thus, the dropout rates were 0%-20%.

In the 3 original telerehabilitation studies, 150 (outpatients in Chile) [25], 35 (outpatients in Greece) [27], and 120 (outpatients in China) [26] participants took part, and 35 (23%), 13 (37%), and 15 (13%) dropped out, respectively.

In the original study that described a long-COVID program, 149 participants (social, health, and care staff in the United Kingdom) [28] took part at the beginning, and only 76 participants took part in all follow-up measurements, which corresponded to a dropout rate of 49%. The original study that conducted qualitative interviews had 11 participants (adults in the United Kingdom) with no dropouts.

### Inclusion Criteria for Postacute COVID-19 Syndrome (Post-COVID/Long-COVID Symptoms)

The inclusion criteria for the studies were patients with a prior COVID-19 infection, either officially diagnosed or self-diagnosed. Additionally, persisting symptoms were a criterion in all studies. Furthermore, some studies focused on specific symptoms.

In the 3 pretest studies, post-COVID/long-COVID symptoms were defined as persisting respiratory symptoms after a COVID-19 infection (>28 days) [20], as persisting symptoms that resulted from a COVID-19 infection [24], and as fatigue symptoms after a COVID-19 infection [4].

In the post-COVID/long-COVID program, participants had to have officially diagnosed or self-diagnosed post-COVID/long-COVID symptoms in line with the National Institute for Health and Care Excellence (NICE) guidelines [28], and in the qualitative interview study, patients who took part in a self-help group on post-COVID/long-COVID symptoms because of their symptoms were approached [29].

### Interventions

**Figure 2:**
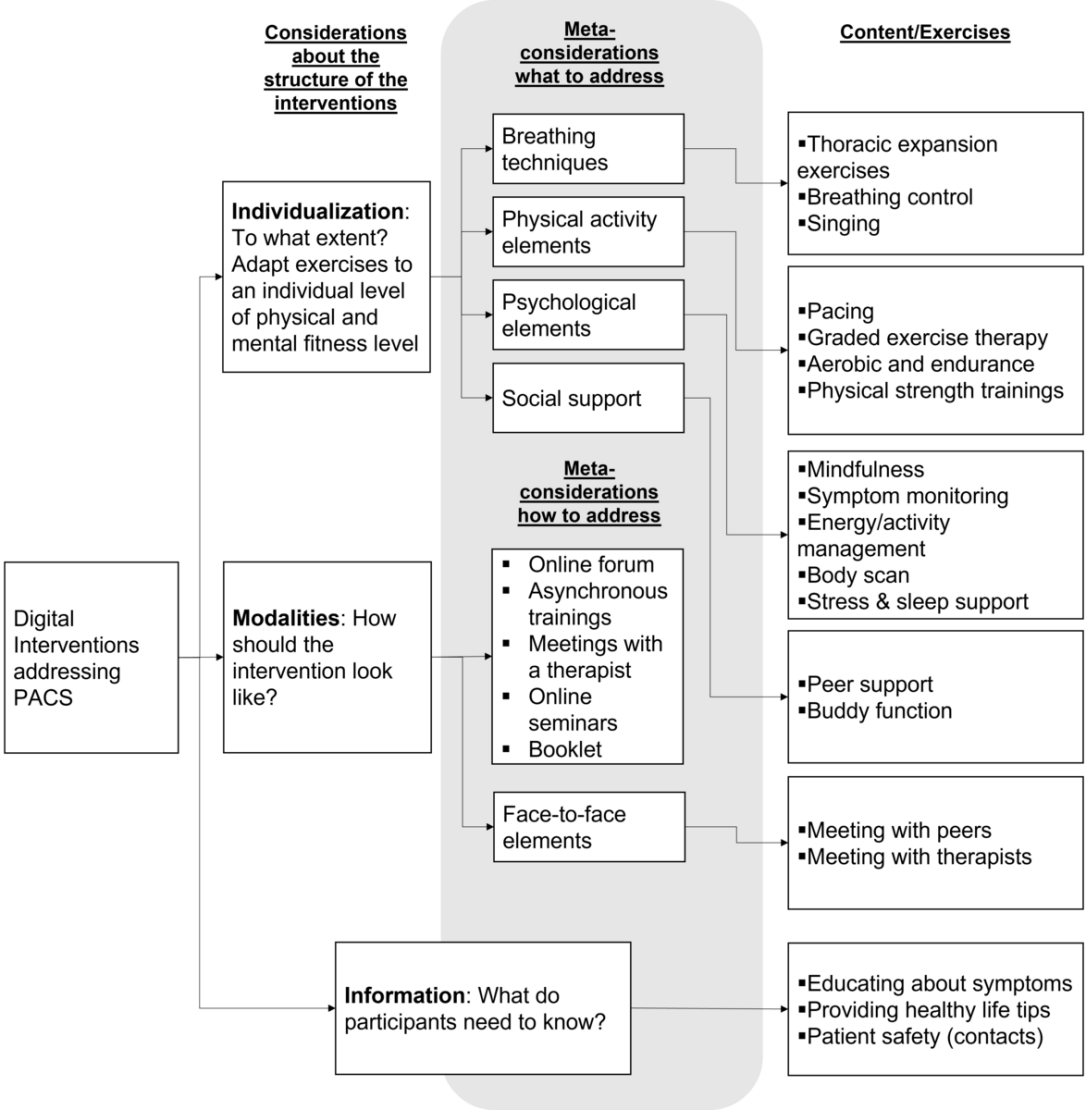
Summary of the interventions carried out in previous literature. PACS: postacute COVID-19 syndrome.

The interventions were dissimilar regarding duration, frequency, social aspects, and general approaches. However, almost all of them used a mixture of psychological and physical exercises or content. A graphical summary of the techniques used can be found in Figure 2.

Specifically, in 1 pretest study, the researchers used a 10-week biweekly breathing and singing class via Zoom (45 minutes). Post-COVID/long-COVID patients received an initial mindfulness and body-scanning exercise, a physical warm-up, vocal and breathing exercises, singing sessions, and nonmandatory online breakout sessions after the classes to support a sense of community [20]. Another pretest study used 3 online meetings with a multidisciplinary (“virtual clinic”) team via Zoom spread over several weeks. Additionally, post-COVID/long-COVID patients received tailored exercises, monitoring, and pacing advice, as well as advice on gradually increasing their physical activity [24]. A further pretest study adopted an existing app (Untire) used to support patients with fatigue caused by cancer. The program described physical activity exercises and aimed to help users manage their energy levels and cognition. Patients were advised to use the app (more than 20 minutes) 3 times a week for more than 2 weeks [4].

One telerehabilitation approach had 24 trainings over 9 weeks with 2 to 3 telerehabilitation sessions each week. The trainings comprised a warm-up (5 minutes), breathing techniques (3 minutes), physiotherapist-taught aerobic and strength exercises (20-30 minutes) with objects and elastic bands, and stretching (5 minutes). Depending on physical conditions, there was higher intensity with higher physical functioning [25]. In another telerehabilitation approach, post-COVID/long-COVID patients were trained on using an eBook in the first phase. Afterwards, they had a 2-month exercise program with daily self-practice and telerehabilitation sessions with a therapist every 10 days. The self-practice exercises comprised breathing techniques, physical exercises (aerobic and body strengthening), relaxation, information about healthy eating, safety instructions, and emergency contact details [27]. A further telerehabilitation approach used a smartphone home exercise program monitored via a chest-worn heart rate device. There were 3 to 4 sessions conducted per week. The exercises comprised breathing control, thoracic expansion, aerobic exercises (ie, walking or running), and limb muscle strength exercises. The level of difficulty and intensity increased over time, whereby the initial intensity was determined by physiotherapists at baseline. Furthermore, teleconsultations were conducted once per week [26].

The post-COVID/long-COVID program used a post-COVID/long-COVID course carried out for 7 weeks with a 1-hour session per week. The sessions were held online via Microsoft Teams and were led by an interdisciplinary team. The intervention comprised educational content as well as relaxation, breathing, and mindfulness exercises. Each session had another topic (eg, understanding COVID-19 or stress management) [28]. Lastly, in the qualitative interview study, patients who used post-COVID/long-COVID self-help groups at their own discretion were interviewed [29].

### Comparison Groups

In most studies, no comparison group was examined [4,20,24,27-29]. Only 2 studies reported comparisons between the intervention group and comparison group (an untreated control group [26] or patients who did/did not receive intensive care [25]). All studies (except one [29]) were longitudinal and compared pre-post intervention measurements.

### Outcome Improvements

Among most papers, an improvement in the perception of physiological health was reported after the digital intervention was adopted. Furthermore, some physical conditions were reported to have improved, and some studies found that fatigue decreased over time. Additionally, some psychological improvements were reported.

Specifically, in 1 pretest study, the authors concluded that fatigue, breathlessness, performance of usual activities, pain/disability, voice quality, and cognition/communication improved [20]. In another pretest study, improvements in health-related quality of life, fatigue, mobility, and the ability to sustain usual activities were reported [24]. Another positive effect of the intervention was that trust in health professionals increased because patients felt that their needs were addressed. In a further pretest study, more than 80% of the participants found some interventions useful, more than 70% liked the exercises and directly applied newly learned strategies, and all participants recommended the intervention for post-COVID/long-COVID patients [4].

Regarding the telerehabilitation format, pre-post improvements were found in the 1-minute sit-to-stand test, the perceptions of health and well-being (measured via the 36-Item Short-Form Health Survey [SF-36]), and fatigue and dyspnea [25]; the 1-minute sit-to-stand test, the perceptions of health and well-being (measured via the SF-36), and anxiety and depression [27]; and the 6-minute walking test, the squat time, and the perceptions of health and well-being (measured via the 12-Item Short-Form Health Survey [SF-12]) [26].

In the post-COVID/long-COVID program, patients reported an increase in their overall health in the pre-post comparison [28]. In the qualitative interview study, patients reported that online peer-support groups helped them to overcome the feeling of inadequate attention by health care professionals, friends, and family. Furthermore, patients reported feeling less alone and more validated after using peer-support groups. Another positive aspect of the intervention was that patients had exposure to a range of symptom management strategies, including exchanging information with patients from other cultures, sociodemographic groups, and countries [29].

### Outcome Nonimprovements

Although assumed, many physiological and psychological functions did not improve through the digital interventions. Specifically, the first pretest study reported that there were no improvements in patient problems regarding the bowel, the bladder, concentration, short-term memory, and unpleasant dreams [20]. In the second pretest study, the researchers stated that due to their small sample, the results were not reliable [24]. Instead, they reported case descriptions. In the third pretest study, less than 50% of the participants found the tips and reminders supportive and motivational. Similarly, fewer than half considered the body exercises useful [4].

The first telerehabilitation found that those who received (compared to those who did not receive) intensive care did not report improvements in their physical ailments, perceived bodily pain, emotional ailments, and perceived mental health through telerehabilitation [25]. Another telerehabilitation program found that patients did not report improvements in their balance, results of the 4-meter self-paced gait speed test, and results of the 5-repetition sit-to-stand test. Furthermore, the intervention could not improve physical activity, heart rate, oxygen saturation, dyspnea, and lower limb fatigue during a 3-minute step test. Similarly, perceived bodily pain, role emotional, and mental health could not be improved either [27]. In the third telerehabilitation study, the authors found that lung function improved similarly among the intervention and control groups. Furthermore, although approximately 90% of the intervention group reported being dyspnea free directly after the intervention (compared to 61.7% in the control group), there were no long-term effects [26].

In the post-COVID/long-COVID program, the authors mentioned that only 30%-50% of the participants reported that their mobility, self-care, usual activities, pain/discomfort, and anxiety/depression improved over time (between before and after the course) and that only 3% of the participants returned to full health [28]. Lastly, the interview study described that patients were unsure about the safety of the suggestions they received from users of the post-COVID/long-COVID peer-support group. Specifically, patients reported that it is difficult to determine how trustworthy the recommendations of other individuals published online are. Moreover, patients reported that sometimes the use of peer-support groups led to negative emotions [29].

## Discussion

### Principal Findings

In summary, digital interventions improved (physiological) health, reduced breathlessness and fatigue, and improved the ability to sustain usual activities. The findings demonstrate the value and feasibility of digital tools for managing post-COVID/long-COVID symptoms, as fatigue is one of the most common symptoms [23,30,31] and digital interventions are generally accepted by society [14]. Accordingly, to relieve fatigue symptoms, mediators, such as physical activity, which were involved in the digital interventions, seem to be important and have been suggested to be effective [24,25]. This is also consistent with recommendations for rehabilitation for outpatients with post-COVID/long-COVID symptoms [32]. However, these findings are contrary to other studies that found that graded physical exercise can be detrimental in treating fatigue [33]. Thus, further research on this matter is urgently needed.

Conversely, all studies found limitations in using digital interventions to manage post-COVID/long-COVID symptoms. For example, the interventions did not improve patients’ problems with the bowel, the bladder, concentration, short-term memory, and unpleasant dreams. Furthermore, the reviewed studies found that only a small percentage of participants reported improvements in mobility, self-care, usual activities, pain/discomfort, and anxiety/depression, and few returned to full health. Thus, the current evidence demonstrates that patients with post-COVID/long-COVID symptoms can benefit from digital interventions to address only a subset of symptoms.

Moreover, it was found that patients might experience cardiac injury after COVID-19 [34]. Considering patient safety, it is suggested to conduct an assessment for post-COVID/long-COVID patients before recommending them to undergo physical activity or exercise [34] or to tailor it according to their needs. Co-creative approaches should be considered, as they harbor additional potential [8].

Patient safety is of importance in medical care and public health. There were 3 original studies regarding telerehabilitation reporting that patients were monitored and measurements of patient safety were applied [24,27,28]. This was however not the case in the online peer-support group study where participants were qualitatively interviewed [29]. Furthermore, 2 interventions provided instructions by trained staff, but the original studies did not elaborate on possible safety measures [20,25]. One study did not mention any safety precautions [4] and one stated that the interventions were conducted unsupervised [26]. Since this topic is important in health settings where no direct contact with a professional health care worker is involved, we recommend that more attention should be paid to this in the future.

Besides physical health, mental health (ie, anxiety and depression) was also addressed in some studies and showed mixed results. In 1 study, anxiety and depression were not assessed [4]. Moreover, 1 study had too few participants to evaluate the change over time [24], and 1 study found no changes in anxiety and depression [20]. In a further study, it was found that 37% of the participants had improved levels of anxiety and depression [28]. Patients also reported that they felt less alone using the online peer-support group, which provides a feasible way to use digital support to decrease loneliness [28]. Three studies found positive effects on mental health [25-27].

These differential effects might relate to the intervention contents and technical features tested in the original studies (Figure 2). However, other factors relating to the research design and methodology might also cause such different results, for example, the sample size or biased sample characteristics (discharged vs nondischarge samples, clinical staff vs individuals from the general population, etc). Five of the eight studies in this review included only a relatively small sample size of fewer than 35 individuals, which might relate to lower statistical power. Thus, we suggest using larger samples to evaluate digital interventions addressing post-COVID/long-COVID symptoms in the future and to run subgroup analyses to find out which interventions work well and which patients need more attention.

It seems that the role of social support for post-COVID/long-COVID patients is important given the fact that many of them do not feel treated and cared for seriously [10]. Although online peer-support groups were found to be helpful in the management and improvement of post-COVID/long-COVID symptoms [29], additional social support is suggested. When designing digital health interventions, the aim should be to help post-COVID/long-COVID patients to self-manage, empower effective self-care, and mobilize social support from health care workers as well as other individuals [8].

### Limitations

There are some limitations that should be considered. First, only a few original studies on the topic have been conducted so far and could be included in this paper, so more future research is required. Second, the duration since the onset of post-COVID/long-COVID symptoms was not included as a control variable in most of the studies. This should, however, be considered since some patients recover a few months later without receiving a specific treatment, while in others, the symptoms become chronic. Therefore, it must be investigated if interventions work similarly in all patients. The findings from studies comparing pre-post intervention measurements might involve time effects besides treatment effects. Third, despite adopting a relatively broad search strategy, some literature might not have been included, especially compared to a review that had a specific medical rehabilitation focus on physical function and dyspnea [17]. In the future, the following search terms should be included in addition to those of this study: telerehabilitation, telemedicine, virtual rehabilitation, computer assisted, home based, and remote [17]. Furthermore, “AND,” “OR,” “/,” “-,” etc should be explicitly applied during the literature search in addition to using leading and plus signs.

Further studies should also address comorbidities as well as blended and mixed intervention effects. Whether participatory and co-creative approaches can more effectively help patients requires much more attention and should be researched systematically in the future with this target group. Lastly, post-COVID/long-COVID symptoms are difficult to compare due to the variety of symptoms and unclear diagnostic criteria. Thus, a nonuniform picture of the results might have occurred because of this circumstance.

### Conclusions

The use of digital interventions is rising when it comes to treating patients with different health impairments, and the initial evidence suggests that this is also the case in post-COVID/long-COVID patients. Current evidence demonstrates the potential for digital interventions to help manage some physical and psychological symptoms. However, the limited research in evaluating the efficacy of digital tools calls for more systematic investigations in addition to evaluating practitioners’ experiences. In further research, theory-based and individualized digital interventions are recommended to better meet the requirements of post-COVID/long-COVID patients, thereby supporting the effective development of digital health services and alleviating the burden on health systems, the society, and individuals.

## Abbreviations

SF-36: 36-Item Short-Form Health Survey
